# Five Cases of Pellagra Treated with Gelsemium

**Published:** 1912-11

**Authors:** Roy Blosser

**Affiliations:** Atlanta, Ga.


### FIVE CASES OF PELLAGRA TREATED WITH GELSEMIUM

By Roy Blosser, M. D., Atlanta, Ga.

So far as I have been able to determine, this drug has not heretofore been used in the treatment of pellagra, probably because it seems to be contraindicated on account of the anemia generally present in this disease.

In endeavoring to relieve a pellagrous patient who complained constantly of severe pain in the feet, (Case No. 1) and as the usual remedies failed to afford satisfactory relief, the author tried gelsemium with rather remarkable results.

In using this drug we must bear in mind its depressing nature, and its effect should be watched closely. It is on record that as little as thirty minims of a tincture caused death in one case of feeble circulation, though it seems possible that this might have been a case of an extreme idiosyncrasy.

The dose used by the writer was three or four drops of a fluid extract, every fourth hour; this is a rather large dose but so far no untoward results have followed its use. An overdose will produce drooping of the eyelids, and in some cases diplopia, but if it is withheld at the appearance of these signs no further unpleasant effects have followed.

Case No. 1. Female, aged 34, married, five children living and well, two children died of diseases of infancy, no miscarriages; family history unimportant. Her past history shows that she probably had syphilis five years ago. She had considerable pelvic trouble for several months before the development of any symptoms that might be considered pellagrous, for which operation was advised by the physician then in attendance, but was not performed.

She was first seen June 18th, 1912. Her history indicated that pellagra began nine months previous with vomiting. According to her statement, she vomited several times every day and sometimes as many as twenty times a day, continuing this for six or seven months. During this time she had an eruption on both forearms (April, 1912). Two months before my first visit to her, the vomiting had abruptly ceased and diarrhoea had set in, with three, twelve or more loose, foul movements a day; about the same time she began to suffer a great deal with pains of a burning character in both feet and from this time on this symptom was the most distressing feature of this case.

Her condition at the first examination was as follows: mucous membrane of tongue and mouth very red and tender; chest and abdomen negative; very much emaciated; mental condition clear, though it was said that she made irrational statements during the night. She had been in bed for the past two weeks on account of weakness and vertigo on attempting to rise. She complained bitterly of the pain in her feet which interfered with sleep and destroyed her appetite. Her weight in good health was 167; the last time she weighed it was 97.

During the following six weeks her condition failed to improve under treatment; she was obviously growing weaker and would take but very little nourishment. Various local applications to the feet failed to give any relief. Internally, bromides and other nerve sedatives gave very slight relief if any at all. She had previously been taking powders containing dionin (a preparation of opium) and she was allowed to continue taking three or four of these a day. Fowler's solution had been given for quite a long time in ascending doses; I gave her hypodermics of soamin every second or third day for four weeks without apparent effect. On the second day of August I stopped all other treatment and began giving the fluid extract of gelsemium, three drops every four hours. In two days the pain in her feet had about stopped and on the fourth day she was able to get up and be dressed. Her appetite returned and she began eating liberally of all knids of food. Her improvement continued uninterruptedly. Her weight, which had gotten down to 82 lbs., has increased 40 lbs., and she seems to be in normal condition, except that her bowels move two or three times a day, though the movements are normal in character, and her feet are apt to ache if she stands too much.

Case N. 2. Female, aged 36, family and past history unimportant. First seen with Dr. Pharr of College Park, Oct. 7, 1912. Her pellagra had begun 18 months previous—the spring of 1911— and had lasted all that summer. She did fairly well last winter, gaining about five lbs. of the 48 previously lost, but her stomach and bowels gave her considerable trouble during this time. Last spring she again became much worse and suffered all summer with severe diarrhoea and with burning pain in stomach and feet. An eruption on hands and arms was quite troublesome both the first and second summer. Various remedies had been tried, including an intravenous injection of salvarsan.

Her condition when we first saw her was as follows:

Patient confined to bed on account of weakness and dizziness. She was very despondent; bowels constipated; she had had occasional spells of vomiting and several times had noticed blood in the vomitus. Examination of chest negative; abdomen exceedingly tender throughout. Appetite very poor. Complains of burning in the feet.

Treatment with gelsemium was begun October 7. She was again seen by Dr. Pharr and myself on October 21. There was noticeable improvement in her mental condition and she was quite cheerful. Only a slight tenderness of the abdomen remained and this was confined to the epigastric region. She was very hungry, but her husband had been afraid to give her any solid food. She was still too weak to leave her bed. After this she was seen about once a week by Dr. Pharr and her improvement, as reported by him, was very satisfactory. On Nov. 16th, she was up, looking after her household affairs, and appeared to have gained considerable flesh though she had not been weighed.

Case No. 3. This woman, aged 48, had suffered from pellagra in a rather severe form for five years. When seen for the first time on Sep. 1, she was not suffering severely, but had a moderate degree of diarrhoea and a good deal of gastric distress; her memory was poor and she could not apply herself well to her work. In two months' treatment with gelsemium these symptoms disappeared and she considered herself well with the exception of a chronic pelvic and kidney trouble which was not thought to have any connection with the pellagra.

Case No. 4. Pellagra of 18 months' duration in a man of 78 years of age. This old man was quite feeble, confined to bed, and was in a peculiar delusional state—constantly trying to get out of bed, pulling of the bed clothes, etc. Frequent attempts to micturate seémed to annoy him greatly.

In three weeks' time the gelsemium had cleared his mental condition so that he does not require constant attention and it has relieved the irritability of the bladder. He now talks quite rationally but is still too weak to leave his bed.

Case No. 5. Female, aged 26. She was seen for the first time with Dr. Pharr three weeks ago. Though she had had undoubted symptoms of pellagra for two years, it had not been severe eough to incapacitate her for household duties.

Dr. Pharr reports that three weeks use of gelsemium has stopped the vomiting—which formerly occurred once or twice a day—and the gastric burning and distress are less troublesome. It is too early to predict what the final outcome of the treatment will be.

---

### MEDICAL COLLEGE DID NOT APPEAR.

The Mandamus case brought by the Southern College of Medicine and Surgery against the Board of Medical Examiners of the State was called for triial in the Superior Court here last week, but there being nobody present for the college except the local attorneys, and they refusing to try the case, it was dismissed by the Court. It will be recalled that this case was brought several months ago to compel the Board of Medical Examiners to examine the graduates of this college. The case was first heard by Judge Morris, was then taken to the Supreme Court by the college, and later came back here for trial before a jury, but as stated above, the college failed to show up and the case was dismissed. The Board refused to examine the graduates of the college because, as the Board claimed, the college was not equipped properly, and was graduating its students before they had completed the course required by law. It is supposed that the dismissal of the case will end the litigation over the matter.

